# Global Infodemiology of COVID-19: Analysis of Google Web Searches and Instagram Hashtags

**DOI:** 10.2196/20673

**Published:** 2020-08-25

**Authors:** Alessandro Rovetta, Akshaya Srikanth Bhagavathula

**Affiliations:** 1 Research and Disclosure Division Mensana srls Brescia Italy; 2 Institute of Public Health, College of Medicine and Health Sciences United Arab Emirates University Al Ain United Arab Emirates

**Keywords:** COVID-19, coronavirus, Google, Instagram, infodemiology, infodemic, social media

## Abstract

**Background:**

Although “infodemiological” methods have been used in research on coronavirus disease (COVID-19), an examination of the extent of infodemic moniker (misinformation) use on the internet remains limited.

**Objective:**

The aim of this paper is to investigate internet search behaviors related to COVID-19 and examine the circulation of infodemic monikers through two platforms—Google and Instagram—during the current global pandemic.

**Methods:**

We have defined *infodemic moniker* as a term, query, hashtag, or phrase that generates or feeds fake news, misinterpretations, or discriminatory phenomena. Using Google Trends and Instagram hashtags, we explored internet search activities and behaviors related to the COVID-19 pandemic from February 20, 2020, to May 6, 2020. We investigated the names used to identify the virus, health and risk perception, life during the lockdown, and information related to the adoption of COVID-19 infodemic monikers. We computed the average peak volume with a 95% CI for the monikers.

**Results:**

The top six COVID-19–related terms searched in Google were “coronavirus,” “corona,” “COVID,” “virus,” “corona virus,” and “COVID-19.” Countries with a higher number of COVID-19 cases had a higher number of COVID-19 queries on Google. The monikers “coronavirus ozone,” “coronavirus laboratory,” “coronavirus 5G,” “coronavirus conspiracy,” and “coronavirus bill gates” were widely circulated on the internet. Searches on “tips and cures” for COVID-19 spiked in relation to the US president speculating about a “miracle cure” and suggesting an injection of disinfectant to treat the virus. Around two thirds (n=48,700,000, 66.1%) of Instagram users used the hashtags “COVID-19” and “coronavirus” to disperse virus-related information.

**Conclusions:**

Globally, there is a growing interest in COVID-19, and numerous infodemic monikers continue to circulate on the internet. Based on our findings, we hope to encourage mass media regulators and health organizers to be vigilant and diminish the use and circulation of these infodemic monikers to decrease the spread of misinformation.

## Introduction

Globally, the internet is an extremely important platform for obtaining knowledge and information about the coronavirus disease (COVID-19) pandemic [1-3]. The Google Trends tool provides real-time insights into internet search behaviors on various topics, including COVID-19 [4]. Social media platforms such as Facebook, Twitter, and Instagram allow users to communicate their thoughts, feelings, and opinions by sharing short messages. A unique aspect of social media data from Instagram is that image-based posts are accessible, and the use of topic-related hashtags allows access to topic-related information for users [5]. In general, there is a growing interest in examining social data to understand and monitor public behavior in real time [6,7].

Research on the internet and social data are called *infodemiology* or *infoveillance* studies [8]. Infodemiology is defined as “the science of distribution and determinants of information in an electronic medium, specifically the Internet, or in a population, with the ultimate aim to inform public health and public policy” [9]. Although several studies have been conducted using infodemiological methods in COVID-19 research, a limited number of studies have examined the extent of COVID-19–related misinformation on the internet [10-14]. We are defining an *infodemic moniker* to be a term, query, hashtag, or phrase that generates or feeds the misinformation circulating on the internet. These monikers can profoundly affect public health communication, giving rise to errors in interpretation, misleading information, xenophobia, and fake news [12-17]. In this context, we aimed to investigate the internet search behaviors related to COVID-19 and the extent of infodemic monikers circulating on Google and Instagram during the current pandemic period.

## Methods

We used Google Trends and Instagram hashtags to explore internet search activities and behaviors related to the COVID-19 pandemic from February 20, 2020, to May 06, 2020. We investigated the following: names used to identify the virus, health and risk perception, lifestyles during the lockdown, and information related to the adoption of infodemic monikers related to COVID-19. The complete list of terms used to identify the most frequently searched queries in Google and hashtag suggestions for Instagram are presented in Multimedia Appendix 1.

The obtained infodemic monikers are characterized as follows:

1. *Generic*: The moniker confuses, due to a lack of specificity.
2. *Misinformative*: The moniker associates a certain phenomenon with fake news.
3. *Discriminatory*: The moniker encourages the association of a problem with a specific ethnicity and/or geographical region.
4. *Deviant*: The moniker does not identify the requested phenomenon.
5. *Other specificities*: We kept two additional points for special cases that prove to be exceptionally serious.

To determine the severity of the various infodemic monikers circulating on the internet, each moniker was assigned 1 to 2 points on the infodemic scale (I-scale) ranging from 0 (minimum) to 10 (maximum). Based on the sum of the I-scale scores, the infodemic monikers were classified as follows: not infodemic (0), slightly infodemic (1), moderately infodemic (2-4), highly infodemic (5-8), and extremely infodemic (9-10).

To assign points, we have adopted the following procedure:

- *Generic*: 1 point is assigned if the keyword is a scientific term but gives rise to misunderstanding (eg, “COVID” instead of “COVID-19”); 2 points are assigned if the keyword is a combination of two scientific terms that can be confused with previously used terms (eg, “SARS-CoV” instead of “SARS-CoV-2” or “SARS-COVID” instead of “SARS-CoV-2” and “COVID-19”).
- *Misinformative*: 1 point is assigned if the keyword can lead to both fake news pointing to individuals (eg, “coronavirus Bill Gates”); 2 points are assigned if the keyword is used to spread misinformation using unrelated or not officially confirmed sources (eg, “coronavirus laboratory”).
- *Discriminatory*: 1 point is assigned if the keyword refers to a specific country and incites unfounded, racial fear (eg, “coronavirus China”); 2 points are assigned if the keywords explicitly target a specific ethnicity (eg, “Chinese coronavirus”).
- *Deviant*: 1 point is assigned if the keyword expresses opinions to influence public opinion (eg, “ban china” or “china app”); 2 points are assigned if the keyword expresses a particular attitude to influence the public (eg, “china puppets” or “savage WHO”).
- *Other specificities*: 1 additional point is assigned when the adoption of a certain moniker is associated with real facts but involves serious health or economic risks (eg, “uv coronavirus”); 2 additional points are assigned when the adoption of a certain moniker involves only health risks (eg, “no sew mask” or “anti-mask protest”).

For each search keyword considered, Google Trends provided normalized data in the form of relative search volume (RSV) based on search popularity scale ranging from 0 (low) to 100 (highly popular). Using these RSV values, we computed the average peak volume (APC) with a 95% CI (for a Gaussian distribution) during the study period.

Instagram, a platform for image-based posts with hashtags, was also screened. We retrieved content based on hashtags through image classifiers every 3-4 days during the study period. All irrelevant content was excluded. The data collected included contents posted on Instagram and self-reported user demographic information. No personal information, such as emails, phone numbers, or addresses, was collected. The data from the Instagram hashtags were collected manually through the Instagram-suggested tags associated with specific countries.

All data used in the study were obtained from anonymous open sources. Thus, ethical approval was not required.

## Results

**Figure 1:**
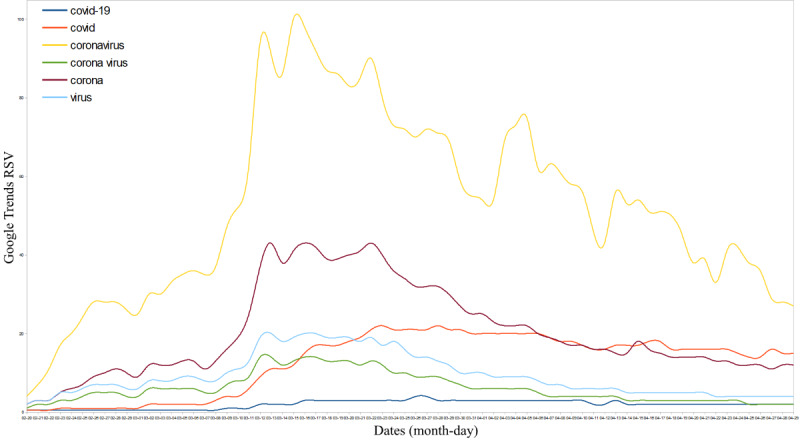
Top scientific and infodemic names related to coronavirus disease (COVID-19) in Google searches globally. RSV: relative search volume.

The top five COVID-19–related infodemic and scientific terms used in Google searches were “coronavirus,” “corona,” “COVID,” “virus,” “corona virus,” and “COVID-19” (Figure 1). The most frequently used keywords globally were “coronavirus” (APC=1378, 95% CI 1246-1537), followed by “corona” (APC=530, 95% CI 477-610) and “COVID” (APC=345, 95% CI 292-398). Several keywords related to COVID-19 were identified (Table 1); of the top 10, four had an I-scale value >4: “corona,” “virus,” “corona virus,” and “coronavirus China.”

**Table 1:** Top infodemic and scientific Google searches related to coronavirus disease (COVID-19) globally.

Keyword	Total average peak volume	95% CI	I-scale^a^ score
coronavirus	1378	1246-1537	4
corona	530	477-610	8
COVID	345	292-398	1
virus	239	212-292	6
corona virus	159	133-186	7
coronavirus Italy	54	45-62	4
COVID-19	53	45-60	0
coronavirus USA	32	29-36	4
coronavirus China	30	25-34	6
coronavirus Germany	23	20-27	4
corona Italy	13	12-14	7
corona Deutschland	12	10-14	7
SARS	9	8-10	5
corona China	9	7-11	9
corona Wuhan	1	0-2	9
SARS-CoV-2	1	0-1	0

^a^I-scale: infodemic scale ranging from 0-10.

**Figure 2:**
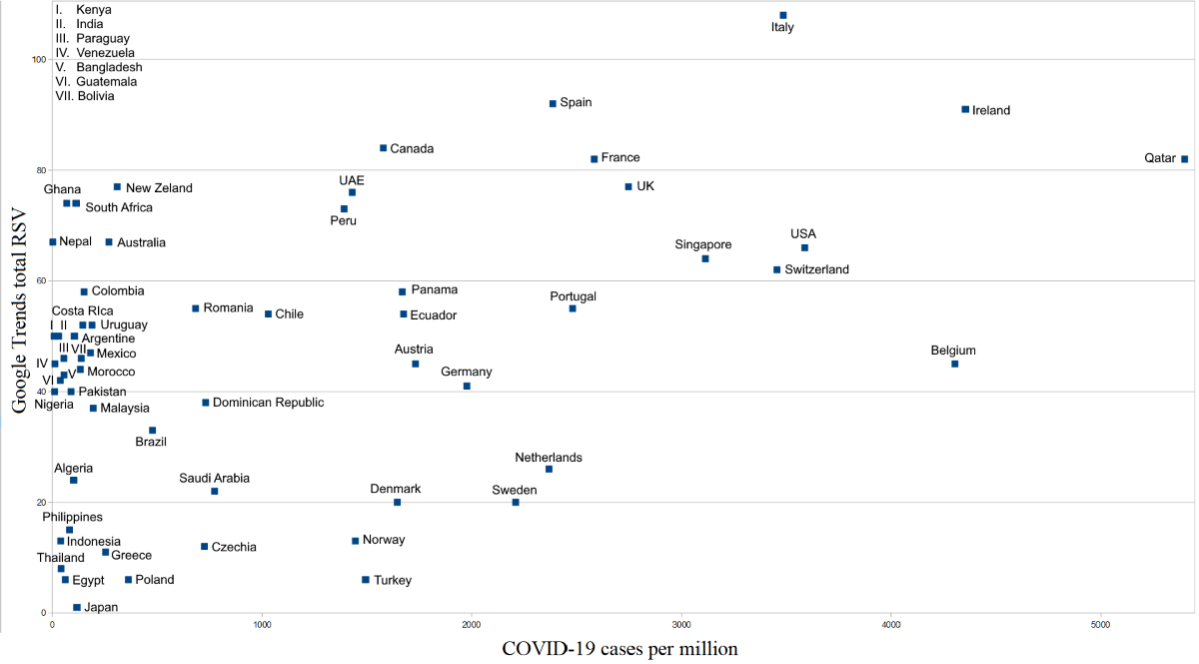
Country-wise dispersion of the scientific and infodemic names of coronavirus disease (COVID-19). RSV: relative search volume.

The country-wise dispersion of the scientific and infodemic names of COVID-19 used in Google searches are shown in Figure 2. Countries with a higher number of COVID-19 cases per 1 million people had greater Google search interest related to COVID-19 (eg, Italy, Spain, Ireland, Canada, France, and Qatar). These COVID-19–related search queries were significantly correlated with the incidence of COVID-19 cases in these countries (Pearson R=0.45, *P*<.001).

**Figure 3:**
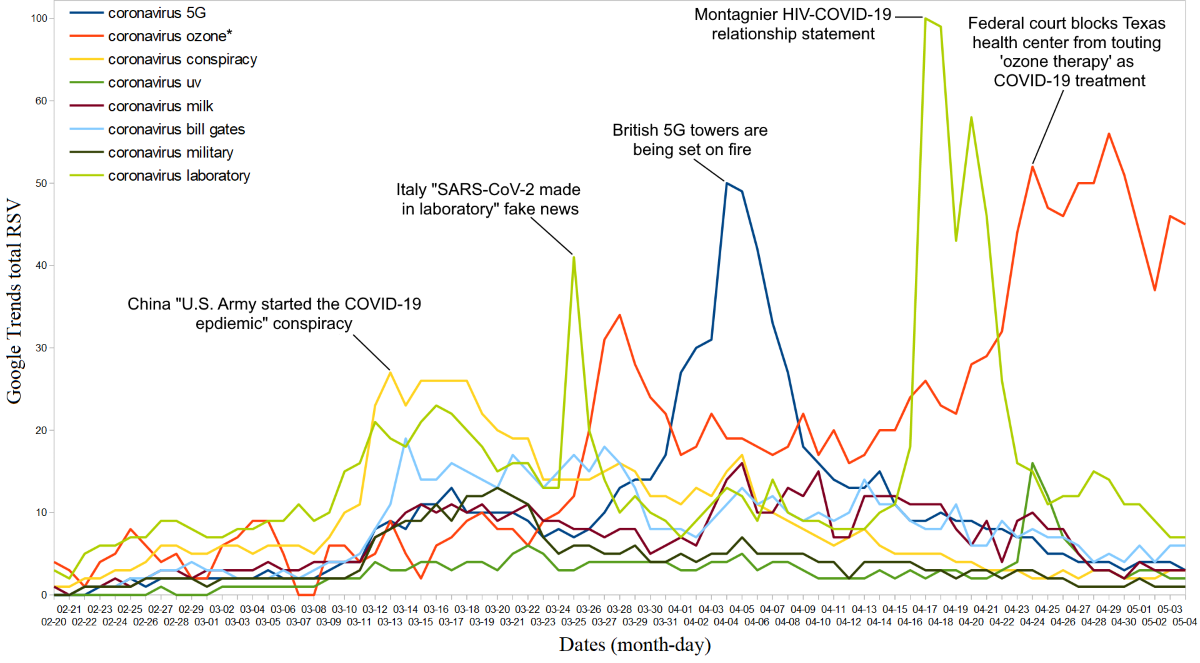
Top global web searches related to coronavirus disease (COVID-19), rated "high" or "extreme" on the infodemic scale, and associated events. RSV: relative search volume.

The top COVID-19 monikers related to fake news (eg, “coronavirus ozone,” “coronavirus laboratory,” and “coronavirus 5G”) that frequently circulated on the internet are presented in Table 2. Among these, “coronavirus conspiracy” and “coronavirus laboratory” had the highest I-scale scores globally (score=9). Additionally, the use of monikers with moderate-to-high infodemicity far exceeded the use of scientific names (Table 1)—52% of Google web searches were moderately infodemic (totalAPC=1487, 95% CI 1326-1647) and 34% highly infodemic (total APC=992, 95% CI 933-1050). The circulation of these infodemic monikers was further examined to understand the events associated with these searches. Infodemic monikers related to coronavirus origins, such as “SARS-CoV-2 made in the laboratory,” went viral (APC=41) when the National Associated Press Agency (Agenzia Nazionale Stampa Associata) of Italy posted a 2015 video about the origins of SARS-CoV-2 virus on March 25, 2020 [18]. In addition, the moniker reached breakout levels (RSV=100) on April 17, 2020, when the French Noble Prize winner Professor Luc Montagnier stated that the new coronavirus was the result of a laboratory accident in a high-security laboratory in Wuhan [19]. Detailed information on the different infodemic monikers and associated events are shown in Figure 3.

**Table 2:** Top global fake news–related Google searches on coronavirus disease (COVID-19).

Keyword	Total average peak volume	95% CI	I-scale^a^ score
coronavirus ozone	19	15-22	5
coronavirus laboratory	16	12-19	9
coronavirus 5G	10	8-13	8
coronavirus conspiracy	9	8-11	9
coronavirus bill gates	8	7-10	6
coronavirus milk	7	6-8	8
coronavirus military	4	4-5	8
coronavirus uv	3	3-4	5

^a^I-scale: infodemic scale ranging from 0-10.

**Figure 4:**
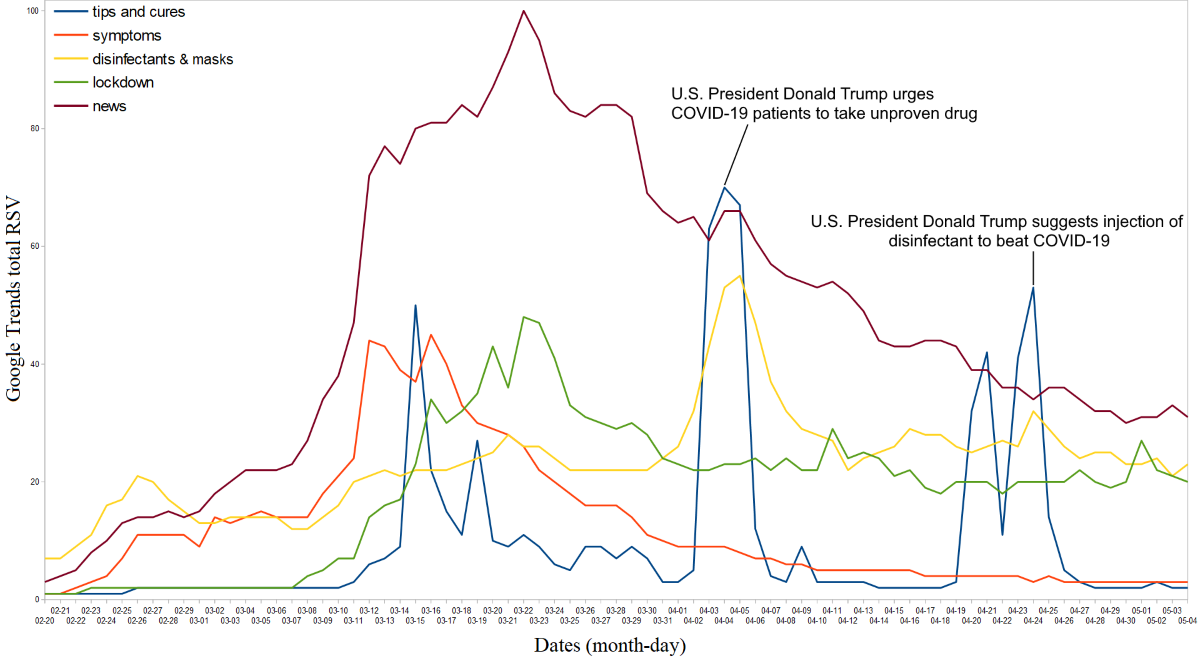
Top global web searches related to health, precautions, and coronavirus disease (COVID-19) news. RSV: relative search volume.

The top searches related to health, precautions, and COVID-19 news are presented in Figure 4. Google searches related to COVID-19 news remained at the top throughout the pandemic period. However, searches related to “tips and cures” for COVID-19 spiked multiple times when the US president suggested that hydroxychloroquine (an unproven drug) was a “miracle cure” on April 4, 2020 (RSV=70) [20]; he also suggested injecting disinfectant to treat COVID-19 on April 24, 2020 (RSV=53) [21]. Other searches related to the use of medical masks and disinfectants (APC=23, 95% CI 21-25), lockdown (APC=19, 95% CI 16-22), and COVID-19 symptoms (APC=12, 95% CI 10-15) were less frequently used in Google searches.

The top five nations most cited by global Instagram users in relation to COVID-19 were Italy (963,000 hashtags), Brazil (551,000 hashtags), Spain (376,000 hashtags), Indonesia (298,000 hashtags), and Turkey (244,000 hashtags) (Table 3). Over 2 million hashtags categorized as extremely infodemic, such as “corona,” “corona memes,” “coronado,” and “corona time,” have been in circulation. Nevertheless, the most globally used hashtags were those related to health and prevention, such as “stay home/safe” and “lockdown life” (>165 million). The contribution of the “covid-19” hashtag for COVID-19–related information was 35.6% (n=26,200,000), followed by “coronavirus” (n=22,500,000, 30.5%), “corona” (n=18,800,000, 25.6%), and “COVID” (n=5,900,000, 8%) (Table 4).

**Table 3:** Top 10 Instagram hashtags related to coronavirus disease (COVID-19), as of May 6, 2020.

Rank	Country	Monikers/hashtags, n^a^	Virus		Health	
			Monikers/hashtags	n^a^	Monikers/hashtags	n^a^
1	Italy	9.63	covid-19	306	health-stay home/safe	933
2	Brazil	5.51	coronavirus	267	lockdown life	718
3	Spain	3.76	corona	188	masks	135
4	Indonesia	2.98	covid	69	memes	25
5	Turkey	2.44	corona memes	14	gym/fitness	24
6	India	1.65	coronavirus Italy	9.63	art/hobbies	22
7	Malaysia	0.89	coronado	8.19	cooking	21
8	Dominican Republic	0.83	corona time	7.12	fashion	16
9	United States	0.75	coronavirus memes	6.41	hair/beard style	14
10	Argentina	0.74	coronavirus Brazil	5.51	fun/party	13

^a^Multiples in 100,000.

**Table 4:** Top Instagram hashtags related to coronavirus disease (COVID-19) scientific and infodemic names.

Hashtag	Count, n (%)
#2019nCOV	38,993 (0.1)
#SARSCoV2	49,011 (0.1)
#SARS	53,633 (0.1)
#COVID	5,890,625 (8.0)
#corona	18,849,998 (25.6)
#coronavirus	22,458,007 (30.5)
#COVID19	26,213,280 (35.6)

## Discussion

### Principal Findings

In light of the ongoing COVID-19 pandemic, we are the first to investigate the internet search behaviors of the public and the extent of infodemic monikers circulating on Google and Instagram globally. Our results suggest that (a) “coronavirus,” “corona,” “COVID,” “virus,” “corona virus,” and “COVID-19” are the top five terms used in the Google searches; (b) countries (eg, Italy, Spain, Ireland, Canada, and France) with a high incidence of COVID-19 cases (per million) have greater Google search queries about COVID-19; (c) “coronavirus ozone,” “coronavirus laboratory,” “coronavirus 5G,” “coronavirus conspiracy,” and “coronavirus bill gates” are widely used infodemic monikers on the internet; (d) although COVID-19 news remains at the top, web searches related to “tips and cures” for COVID-19 spiked when the US president speculated about a “miracle cure” and the use of a disinfectant injection to treat COVID-19; (e) 66.1% (n=48,700,000) of Instagram users used “COVID-19” and “coronavirus” as a hashtag to disperse information related to COVID-19.

Exploring research using nontraditional data sources such as social media has several implications. First, our results demonstrated a potential application for the use of Instagram as a complementary tool to aid in understanding online search behavior; we also provided real-time tracking of infodemic monikers circulating on the internet. The strength of this study is the investigation of various infodemic monikers dispersed on the internet and correlating them with the events associated with that particular day. Although we used correlations to examine the possible linear association between search queries and the event, it should be noted that use of a search engine is voluntary and self-initiated search queries represent the users who are truly curious or worried about a situation. Thus, we believe that the unobtrusive search behavior of netizens may have resulted in an increase in search volume. By characterizing and classifying various infodemic monikers based on the degree of infodemicity (ie, via the I-scale), researchers can foster new methods of using social media data to monitor the monikers' outcomes. The analysis and methods used in this study could aid public health and communication agencies in identifying and diminishing infodemic monikers circulating on the internet.

Findings from this study validate and extend previously published works that used Google keywords [1,12,13]. We also demonstrate the potential for the use of Instagram hashtags to monitor and predict both the cyber behavior and relaying of misinformation on the internet [22-24]. In 2017, Guidry et al [22] studied Ebola-related risk perception in Instagram users and identified that a significant proportion of posts had rampant misinformation about the Ebola disease during the outbreak. In addition, the percentage of Instagram posts and tweets posted by health organizations (eg, Centers for Disease Control and Prevention, World Health Organization, Médecins Sans Frontières [Doctors Without Borders]) that correct misinformation is less than 5% [22]. In general, negative information posted on the internet tends to receive a greater weight among netizens. Thus, this should be counter-balanced with evidence-based content from health organizations, particularly in the current pandemic situation. For example, when the US president suggested injecting disinfectant to treat COVID-19, the number of Google searches considering it as a cure sharply increased (APC=53) and resulted in 30 cases of disinfectant poisoning within 18 hours in New York City [25]. Health authorities should be vigilant and provide more positive and informative messages to combat the circulation of infodemic monikers on social media. Future studies will need to investigate the influence of infodemic monikers on individual cyber behavior.

### Limitations

Our study used Google Trends, which only provides the search behavior of people using the Google search engine. Furthermore, our study focused on Google and Instagram for data retrieval. Future studies should consider studying the same topic on other social media platforms to capture a more diverse population of users. Instagram searches were conducted manually, introducing a potential for human error. Going forward, the use of an automated program can improve the accuracy of the data collected and analyzed. Lastly, Google Trends did not provide any information about the methodology used to generate search data and algorithms.

### Conclusion

Using Google Trends and Instagram hashtags, the present study identified that there is a growing interest in COVID-19 globally and in countries with a higher incidence of the virus. Searches related to “COVID-19 news” are quite frequent and two thirds of Instagram users have used “COVID-19” and “coronavirus” as hashtags to disperse information related to the virus. Several infodemic monikers are circulating on the internet, with “coronavirus conspiracy” and “coronavirus laboratory” identified as the most dangerous (I-scale score=9). Given the prevalence of infodemic monikers, mass media regulators and health organizers should monitor and diminish the impact of these monikers. These governing bodies should also be encouraged to take serious actions against those spreading misinformation in social media.

## Appendix

Multimedia Appendix 1 Terms used to identify the most frequently searched queries in Google and the hashtag suggestions for Instagram.

## Abbreviations

APC: average peak volume
COVID-19: coronavirus disease
I-scale: infodemic scale
RSV: relative search volume
