# Supplementary material for: Global Infodemiology of COVID-19: Analysis of Google Web Searches and Instagram Hashtags
Source: J Med Internet Res. 2020 Aug 25;22(8):e20673. doi: 10.2196/20673 (PMC7458585; doi:10.2196/20673)
Supplement: Multimedia Appendix 1 [file jmir_v22i8e20673_app1.docx]

**Multimedia Appendix 1**

**Google Trends**

All keywords reported here are those that showed average peak volumes > 1. The keywords selection was done through Google Trends “rising” and “top” queries.

**Virus denominations:** coronavirus, covid, covid-19, coronavirus covid-19, sars, sars-cov-2, corona virus, corona, virus, вирус, coronavirus italy, coronavirus italia, coronavirus lombardia, coronavirus italie, coronavirus italien, corona virus italia, coronavirus china, coronavirus china, coronavirus cina, coronavirus chine, coronavirus chinese, corona wuhan, coronavirus wuhan, corona china, covid china, covid 19 china, corona deutschland, covid deutschland, coronavirus usa, coronavirus united states, coronavirus america, coronavirus estados unido, coronavirus allemagne, coronavirus france, covid france, coronavirus pandemic, covid pandemic, covid 19 pandemic, pandemia, пандемия

**Coronavirus laboratory group:** montagnier, coronavirus leonardo, coronavirus laboratory, coronavirus hiv, hiv covid, hiv covid 19, covid-19 laboratory, laboratory coronavirus, laboratorio coronavirus, covid laboratory, covid 19 laboratory, corona hiv

**Coronavirus milk group:** leche coronavirus, coronavirus leite, coronavirus milk, coronavirus milch, latte coronavirus, covid milk, covid 19 milk, covid-19 milk

**Coronavirus ozone group:** coronavirus ozone, ozono, ozone, coronavirus ozone, ozone virus, covid ozone, covid ozono

**Other infodemic keywords:** coronavirus 5g, covid 5g, covid 19 5g, coronavirus bill gates, covid bill gates, coronavirus military, coronavirus militares, covid military, covid 19 military, coronavirus trump, trump tips, covid trump, covid 19 trump, coronavirus conspiracy, coronavirus uv, disinfectant injection

**Lockdown:** quarantena, lockdown, quarantine, Quarantäne, cuarentena, quarantane, карантин, coronavirus lockdown, covid lockdown, coronavirus quarantena, covid 19 lockdown, lockdown 2020, карантин

**Tips & cures:** coronavirus cure, coronavirus vaccine, coronavirus treatment, cura coronavirus, coronavirus tips, dicas coronavirus, coronavirus cure, coronavirus vaccine, coronavirus treatment, cura coronavirus, коронавирус вакцина

**Symptoms:** coronavirus symptoms, sintomas de coronavirus, sintomas coronavirus, coronavirus symptome, corona symptoms, sintomas corona, corona Symptome, коронавирус симптомы

**Disinfectant & mask:** disinfectant, disinfettante, Desinfektionsmittel, desinfectante, disinfectant spray, amuchina, mask, mascherina, maske, mascara, ffp3, ffp2, коронавирус вакцина

**News:** coronavirus deaths, coronavirus cases, covid cases, covid deaths, covid-19 cases, coronavirus news, covid news, noticias coronavirus, ultimas noticias coronavirus, coronavirus update, коронавирус Новости, coronavirus hoy, covid hoy, covid 19 updates, covid 19 update

**Instagram**

All the keywords shown are those that exceed 50,000 tags. The keywords selections was made through Instagram hashtags suggestions

**Coronavirus:** coronavirus, coronavìrus, coronavirusoutbreak, coronaviruspandemic coronavirusmemes, coronavirusespana, coronavirus, coronavirusbrazil, coronavirususu, coronavirusmemes, coronavirusoutbreak, coronavirusitalianews, coronavirusitalia, coronavirusu, coronavairus, coronamemes, coronatime, coronaturkiye, coronaextra, coronaindonesia, coronavirusitaly, corona, coronavirusbrazil, coronaviruspain, coronavirusindonesia, coronavirusturkey, coroanvirusindia, coronavirusmalasiya, coronavirusdominicanrepublic, coronavirususa, coronavirusus, coronavirusargentina, coronavirusunitedkingdom, coronavirusuk, coronavirusdr, coronavirusargentina, coronavirusfrance, coronaviruspagna, coronaviruspandemic, covidpandemic, covid19pandemic

**COVID:** covidkindness, covidart, covidmuseum, covidcooking, covidphotodiaries, covidiot, covidiots, covidmemes, covidmask, coronavid19, coronavirus2020

**COVID-19:** covid_19, covid19, covid19indonesia, covid2019, covid-19, covid19italia, covid_19, covid2020, covid19italia, covid19us, covid19_us, covid19

**SARS:** sars

**SARS-COV-2:** sars-cov-2

**All > 100,000 utilized hashtags suggestions related to:** lockdown, lockdown life, lockdown art, lockdown hobbies, lockdown cooking, lockdown fashion, lockdown games, lockdown tv, lockdown videos, lockdown gym, lockdown activities, lockdown sport, lockdown fun, quarantine, quarantine life, quarantine art, quarantine hobbies, quarantine cooking, quarantine fashion, quarantine games, quarantine tv, quarantine videos, quarantine gym, quarantine activities, quarantine sport, quarantine fun, stay home, stay safe, stay healthy, stay strong, covid memes, covid-19 memes, corona memes, coronavirus memes, virus memes, corona virus/beer memes, coronavirus life, coronavirus art, coronavirus hobbies, coronavirus cooking, coronavirus fashion, coronavirus , coronavirus games, coronavirus tv, coronavirus videos, coronavirus gym, coronavirus activities, coronavirus sport, coronavirus fun, coronavirus precautions, corona, corona life, corona art, corona hobbies, corona cooking, corona fashion, corona, corona games, corona tv, corona videos, corona gym, corona activities, corona sport, corona fun, corona precautions, covid, covid life, covid art, covid hobbies, covid cooking, covid fashion, covid, covid games, covid tv, covid videos, covid gym, covid activities, covid sport, covid fun, covid precautions, virus life, virus art, virus hobbies, virus cooking, virus fashion, virus , virus games, virus tv, virus videos, virus gym, virus activities, virus sport, virus fun, virus precautions covid-19, covid-19 life, covid-19 art, covid-19 hobbies, covid-19 cooking, covid-19 fashion, covid-19, covid-19 games, covid-19 tv, covid-19 videos, covid-19 gym, covid-19 activities, covid-19 sport, covid-19 fun, covid-19 precautions, medical masks, hygiene, coronavirus 5g, covid 5g, covid-19 5g, corona 5g, coronavirus laboratory, corona laboratory, covid laboratory, covid-19 laboratory, sars laboratory, sars-cov-2 laboratory, virus laboratory, coronavirus nation, corona nation, covid nation, covid-19 nation, sars nation, sars-cov-2 nation, virus nation, coronavirus pubclic figures, corona public figures, covid public figures, covid-19 public figures, sars public figures, sars-cov-2 public figures, beer, coronavirus beer, corona beer, covid beer, covid-19 beer, sars beer, sars-cov-2 beer, coronavirus cure, corona cure, covid cure, covid-19 cure, sars cure, sars-cov-2 cure.
